# Overexpression of OCT4 is associated with gefitinib resistance in non-small cell lung cancer

**DOI:** 10.18632/oncotarget.12999

**Published:** 2016-10-31

**Authors:** Bin Li, Zhouhong Yao, Yunyan Wan, Dianjie Lin

**Affiliations:** ^1^ Department of Respiratory Medicine, Shandong Provincial Hospital Affiliated to Shandong University, Shandong University, Jinan, Shandong, China, 250021

**Keywords:** NSCLC, chemoresistance, OCT4, gefitinib, proliferation, apoptosis

## Abstract

Epidermal growth factor receptor (EGFR)-targeted tyrosine kinase inhibitors (TKIs) have emerged as first-line drugs for non-small cell lung cancers (NSCLCs). However, the resistance to TKIs represents the key limitation for their therapeutic efficacy. We found that the difference of OCT4 expression between NSCLC and the adjacent non-tumourous tissues was statistically significant. Knockdown of OCT4 in NSCLC cells could decrease cell proliferation, and potentiate apoptosis induced by gefitinib, suggesting OCT4 may contribute to gefitinib resistance in NSCLC.

## INTRODUCTION

Non-small-cell lung cancer (NSCLC) is seen with yearly increased mortality in the Asia-Pacific region. Surgery is considered the most effective treatment for NSCLC patients [1]. But a great number of the patients with NSCLC present advanced-stage of disease at primary diagnosis. Albeit growing development in clinical and foundation oncological researches, the prognosis of advanced NSCLC remains unsatisfied yet, of which a 5-year overall survival rate is only 11% or so [2–3]. According to the reports in the past decades, tyrosine kinase inhibitors(TKIs) targeted to epidermal growth factor receptor(EGFR) may significantly reduce mortality rate of patients with NSCLC [4–5]. Nevertheless, the great majority of patients received EGFR-TKIs therapeutic strategy eventually become tolerant. Understanding these mechanisms is essential for advanced NSCLC treatment.

As a member of the POU transcription factor family, the octamer-binding protein 4 (OCT4) implicated in proliferation and differentiation of cell [6]. dysregulation of OCT4 appears to be involves in several important processes during tumorigenesis, tumor progression and even chemoresistance [7–8]. Moreover, some study had pinpointed that OCT4 was not only a desired prognostic factor, but also a potential target for therapies in theriomas [9–10]. However, little is cleared about whether not OCT4 plays functional role in EGFR-TKIs resistance of NSCLC and its underlying mechanism. Here we investigated the relationship between OCT4 and acquired drug-resistance in these NSCLC patients received EGFR-TKIs therapy.

## RESULTS

### OCT4 expressions is significantly upregulated in NSCLC

**Figure 1:**
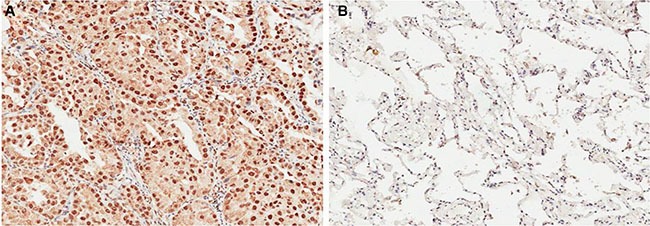
(**A**) Immunohistochemical analysis revealed that NSCLC tissue had higher OCT4 expression; (**B**) Immunohistochemical analysis revealed that corresponding adjacent noncancerous tissues had lower OCT4 expression.

**Figure 2:**
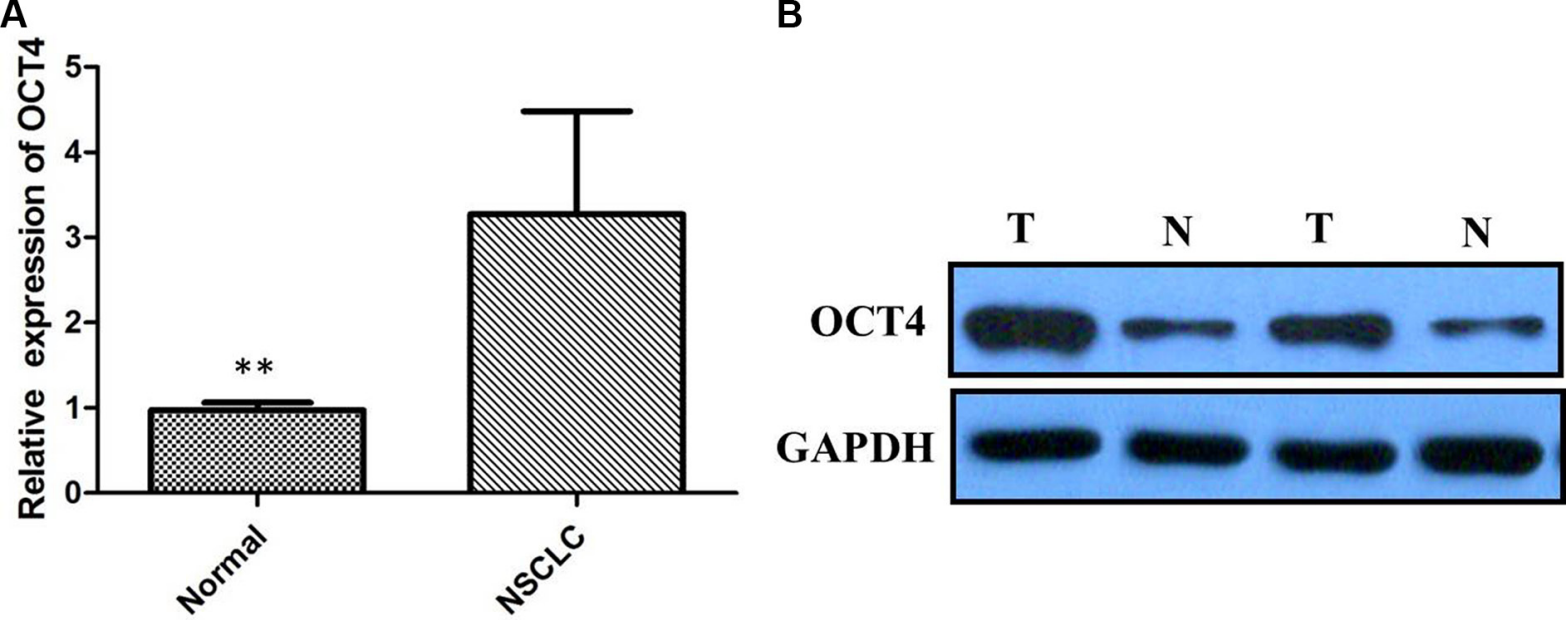
(**A**) Quantitative real-time PCR showing expression level of OCT4 mRNA in NSCLC tissues; (**B**) Western blots showing expression of OCT4 protein in NSCLC tissues;

We detected OCT4 expression level by IHC in 86 NSCLC cancer and premalignant tissues. We found that 70.93% (61/86) of NSCLC tissues presented positive staining for OCT4, whereas high expression of OCT4 was observed in 31.40% (27/86) of precancerous tissues, the difference of OCT4 expression was statistically significant (Figure 1A and 1B, *P* < 0.001), indicating that OCT4 have a great importance at aspect of tumorigenesis in NSCLC. Furthermore, qRT-PCR and western blot analysis were applied to detect mRNA and proteins level in the same samples used for IHC. Results revealed that compared to peritumoral tissues, expression level of OCT4 mRNA and protein in tumor tissues were significantly higher (*p* < 0.01, Figure 2A and 2B), determining that OCT4 may have an essential role in occurrence of NSCLC.

### Involvement of OCT4 expression in EGFR-TKI-resistant cells

**Figure 3:**
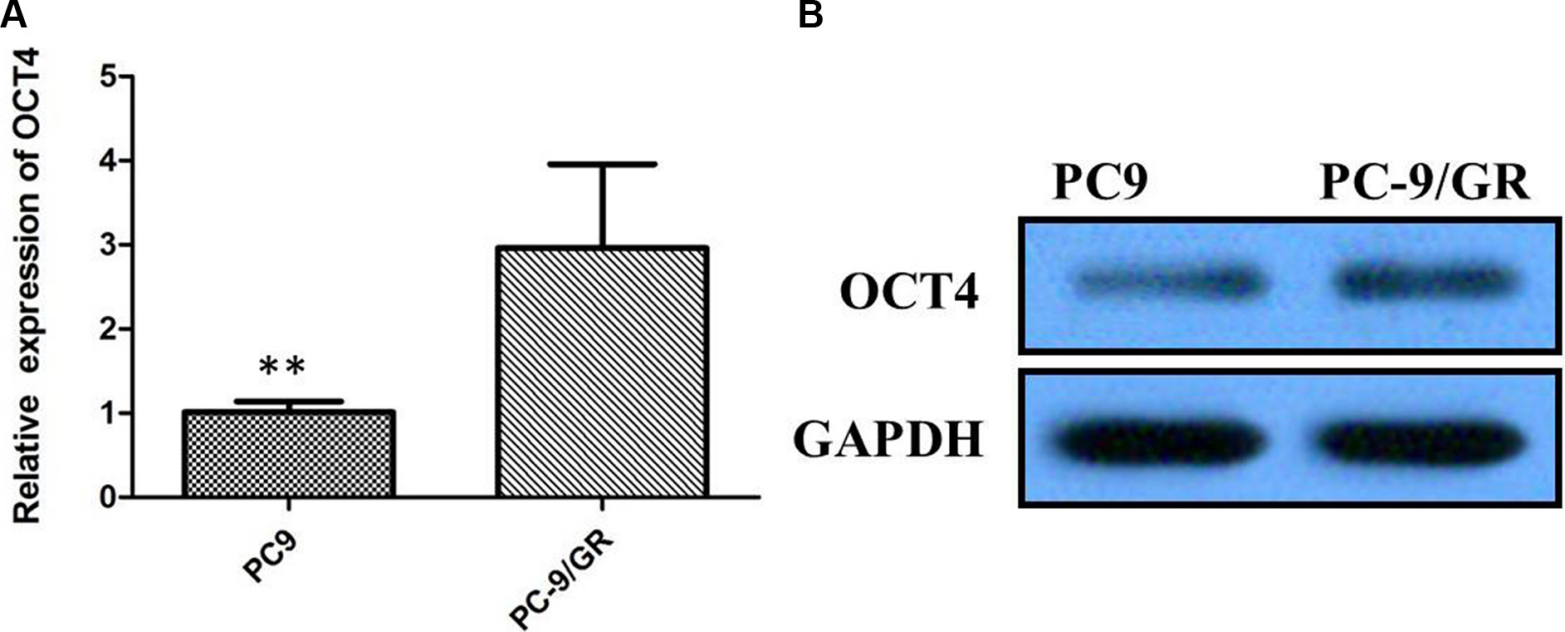
(**A**) Quantitative real-time PCR showing expression level of OCT4 mRNA in PC-9 and PC-9/GR cells; (**B**) Western blots showing expression of OCT4 protein in PC-9 and PC-9/GR cells;

To investigate whether not OCT4 involves in modulation of TKI sensitivity, PC-9 cell line sensitive to TKI and PC-9/GR resistant to gefitinib were applied to examine the levels of OCT4 by qRT-PCR. Suggested by results, more expression level of OCT4 was detected in PC-9/GR cells in comparision with PC-9 cells (*P* < 0.01; Figure 3A). Similarly, overexpression of OCT4 protein was detected in PC-9 GR cells in comparision with PC-9 cells by western blot analysis (Figure 3B).

### OCT4 might affect the proliferation ability of NSCLC *in vitro*

**Figure 4:**
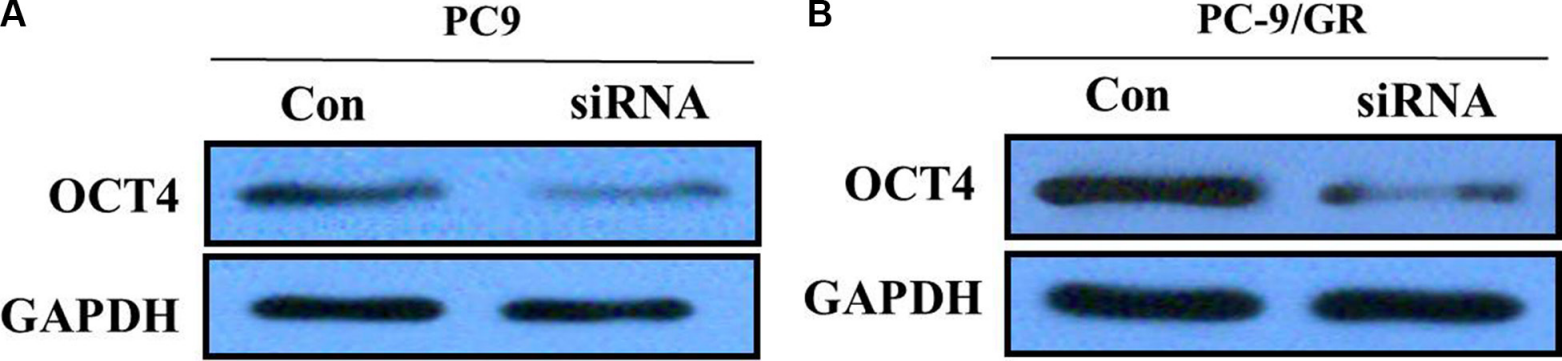
(**A**) Western blots showing knocking-down of OCT4 in PC-9 cells; (**B**) Western blots showing knocking-down of OCT4 in PC-9/GR cells;

**Figure 5:**
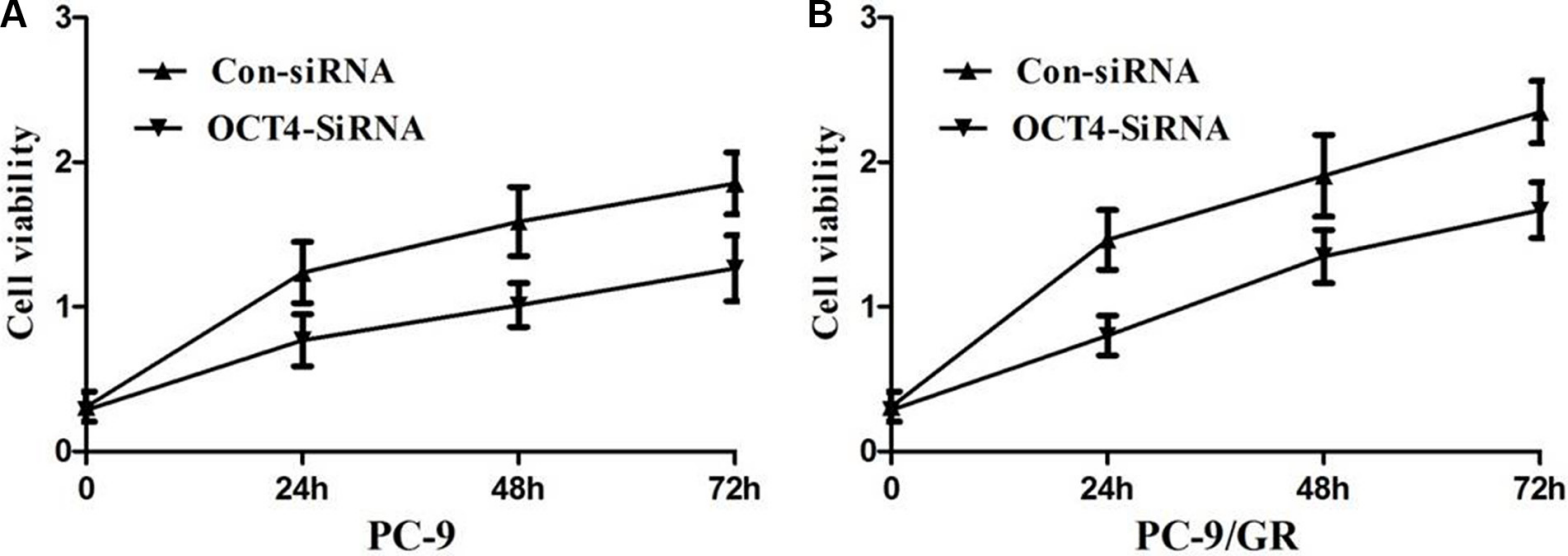
(**A**) MTT assay showing knocking-down of OCT4 markedly suppressed the ability of proliferation of PC-9 cells; (**B**) MTT assay showing knocking-down of OCT4 markedly suppressed the ability of proliferation of PC-9/GR cells;

To further test whether not OCT4 affects proliferation of NSCLC cells, we exclusively silenced the OCT4 gene expression in PC-9 and PC-9/GR cells by exclusive siRNA. After that, expression level of OCT4 proteins was significantly decreased in PC-9 and PC-9/GR cells (Figure 4). Then, We explore the effect of OCT4 knockdown on the proliferation of PC-9 and PC-9/GR cells. MTT assay demonstrated that OCT4 silencing could decrease cellular activities of PC-9 and PC-9/GR cells (Figure 5A and 5B), possibly suggesting that OCT4 levels was tightly correlated with NSCLC cells growth.

### Gefitinib induced apoptosis in the OCT4-deficient-PC-9/GR cell line

**Figure 6:**
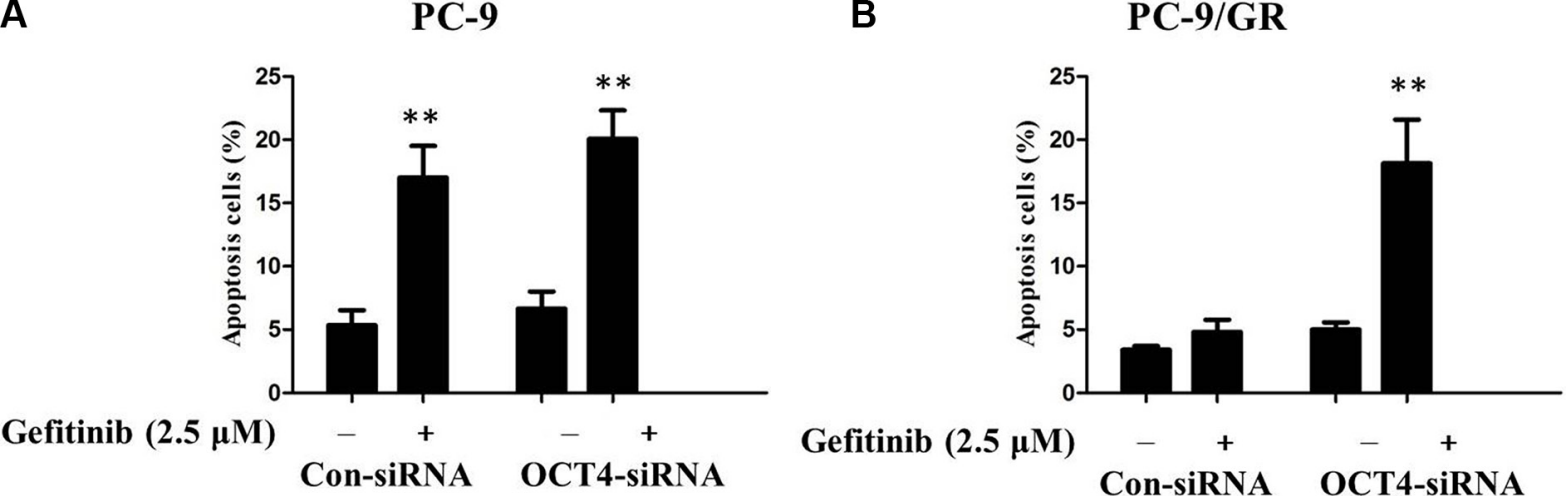
(**A**) Gefitinib induced signicantly potentiated apoptosis in PC-9 cells transfected with either OCT4 siRNA or control; (**B**) CTSL silencing signicantly potentiated apoptosis induced by gefitinib in PC-9/GR with OCT4 knockdown compared with control.

To clarify how gefitinib induced apoptosis in the OCT4-deficient-cell line sensitive to gefitinib, flow cytometry analysis was employed to detect cell apoptosis. Therefore, control siRNA and OCT4 siRNA were transfected into PC-9 and PC-9/GR cells respectively, which were subsequently dealt with or without gefitinib treatment (2.5 μM) for 48 h. The results showed that gefitinib induced significantly apoptosis in PC-9 cells transfected with either OCT4 siRNA or negative control (Figure 6A). Interestingly, knockdown of OCT4 remarkably enhanced apoptosis of PC-9/GR cells received gefitinib in comparision with matched group, suggesting OCT4 can positively induce gefitinib resistance in NSCLC cells. That is to say, knockdown of OCT4 can reverse gefitinib resistance and promote apoptosis (Figure 6B). But for sensitive PC-9 cells, OCT4 silencing doesn't make a difference for gefitinib treatment.

## DISCUSSION

NSCLC is the primary factor of cancer mortality around the world. Hence, there is a pressing need of novel therapeutic strategies due to their relative insensitivity to chemotherapy [11]. The high incidence of EGFR tyrosine kinase domain mutation in NSCLCs has provided the rationale for successful application of a class of EGFR-TKIs for first-line NSCLC treatment [12]. Sad to say, due to lack of effective therapy to block or reverse acquired chemoresistance, nearly all patients suffer from relapse, even life-threatening [13–14]. Therefore, it is of great urgency to find its symbolic marker in order to have a better understanding of its mechanism and development stages, which is likely to further the finding of effective therapeutic targets and prognostic indicators. The data we present here suggest that OCT4 promotes gefitinib resistance in NSCLCs.

As a transcription factor, OCT4 is capable of keeping versatility and self-renewing of embryonic stem cells through combining with an octameric consensus sequence to activate its target genes [15–18]. Over-expression of OCT4 is found in lung cancer-derived CD133- and CD44-positive cells, anticancer drug-selected breast cancer cells [18–22], which exhibit enhanced resistance to chemotherapeutic agents. In our research, we firstly determined the OCT4 level in 86 cases of NSCLC, and results revealed that compared to peritumoral tissues, expression level of OCT4 mRNA and protein in tumor tissues were significantly higher. IHC staining showed overexpression of OCT4 was observed in 70.93% (61/86) of NSCLC specimens when compared with adjacent non-neoplastic tissues (31.40%, 27/86), the difference of OCT4 expression was statistically significant. Moreover, more expression of OCT4 was observed in the gefitinib insensetive PC-9/GR cell compared with the gefitinib sensitive PC-9 cell, indicating that OCT4 is a possible tumorgenesis contributor in NSCLC.

To further probe into its potential mechanism, we knockdown the OCT4 expression by siRNA in PC-9/GR and PC-9 cell model. We noticed that growth of PC-9/GR and PC-9 cells were evidently inhibited followed by knockdown of OCT4, indicating that OCT4 proteins were correlated positively with the proliferation of NSCLC cells *in vitro*. Furthermore, knockdown of OCT4 remarkably enhanced apoptosis of PC-9/GR cells received gefitinib in comparision with matched group, suggesting OCT4 contributes to gefitinib resistance in NSCLC cells and that knockdown of OCT4 remarkably enhanced gefitinib-induced apoptosis of PC-9/GR cells. The reason why knockdown of OCT4 can inhibit proliferation and induce apoptosis is that it targets to numerous regulators related to these biological pathways.

In summary, our results suggest that OCT4 functions as a carcinogenic factor in human NSCLC. In both of NSCLC and gefitinib resistant cells, OCT4 expression level was significantly increased. OCT4 silencing could reverse resistant phenomenon of gefitinib in NSCLC cells. Accordingly, OCT4 may be a potential target spot for the treatment of NSCLC.

## MATERIALS AND METHODS

### Clinical specimens

Eighty-six paired NSCLC tissues and matched adjacent normal tissues were obtained from Shandong Provincial Hospital Affiliated to Shandong University between 2010 and 2015. All patients recruited in this study were not subjected to preoperative radiotherapy and/or chemotherapy and were diagnosed as infiltrating carcinoma by pathologists. For all patients, histological type and grade of cancer cell differentiation were reevaluated and determined by the classification system of the World Health Organization modified in 2004, and postsurgical pathological staging was determined based on the international staging system. Tumor specimens and corresponding adjacent normal tissues were collected and stored in liquid nitrogen until use. The study was approved by the Medical ethics committee of Shandong Provincial Hospital Affiliated to Shandong University.

### Cell culture and reagents

The human PC9 and PC9 gefitinib-resistance (PC-9/GR) lung adenocarcinoma cell lines were cultured in RPMI and DMEM, respectively, supplemented with 10% fetal bovine serum, 100 units/ml penicillin, and 100 mg/ml streptomycin at 37°C in a humidified environment containing 5% CO_2_. N-methyl-N’-nitro-N-nitrosoguanidine (MNNG) and was continuously subcultured with 0.2 μmol/L of gefitinib for an additional 6 months. Gefitinib were provided from Astrazeneca (Alderley Park, UK). Gefitinib was prepared in dimethyl sulfoxide (DMSO) to obtain a stock solution of 10 mM.

### TMA and IHC

TMA blocks consisting of a representative tumor core section 2 mm in diameter from each formalin fixed paraffin block (SuperBioChips Laboratories, Seoul, Republic of Korea) were manufactured for IHC analysis. IHC staining was performed using the BenchMark XT Slide Preparation System (Ventana) and rabbit polyclonal anti-OCT4 antibody (1:100, Abcam, Cambridge, MA, USA). Assessments of the staining were evaluated under a light microscope by two experienced pathologists who did not know the exact condition of the patient and the scores were depended on staining intensity and proportion as previously described. For each tissue core, the intensity of staining was categorized as follows: 0, negative; 1 weak; 2 moderate; and 3, strong. And based on the proportion of staining, the degree was scored on a scale of 0 (< 5%, absent), 1 (5%–25%, sporadic), 2 (25%–50%, focal) and 3 (> 50%, diffuse). The final score of each staining was obtained by multiplying the two scores. The IHC score ranged from 0 to 9, which less than 4 points was determined to negativity.

### Quantitative reverse transcription polymerase chain reaction (qRT-PCR)

Real-time PCR analyses were performed with SYBR Premix ExTaq II kit (Takara, Dalian China). Results were normalized to the expression of GAPDH. Forward and reverse primers for OCT4 5′-TGAAGCTGGAGAAGGAGAAGCTG-3′ (forward) and 5′-TCTTTCTGCAGAGCTTTGATGTCCT-3′ (reverse). The qRT-PCR assays and data collection were performed on ABI 7500, and results were analyzed and expressed relative to threshold cycle values (ΔCt), then converted to fold changes using the 2−ΔΔCt method. GAPDH was used as an internal control.

### Western blot

Cells were treated with different concentrations of gefitinib. Western blot was performed as described elsewhere. Primary antibodies used were as follows: OCT4 (Abcam, Cambridge, MA, USA), GAPDH (Cell Signaling Technology, Beverly, MA, USA). Proteins were visualized with a horseradish peroxidase-coupled secondary antibody from Cell Signaling Technology. Membranes were then washed again three times for 10 min each with TBS-T. Target protein bands were visualized using the enhanced chemiluminescence method. All western immunoblot analyses were performed three times.

### Transfection

RNA duplexes were obtained from the Ribobio Company (Guangzhou, PR China). The strand sequences of siRNA for human OCT4 was 5′-AAGGAUGUGGUCCGAGUGUGG-3′. Cells cultured in 6-well plates were transfected with 50 nM siRNA duplexes per well using the Lipofectamine 2000 reagent (Invitrogen, Carlsbad, CA, USA) prepared in DMEM basic medium. SiRNA/Lipofectamine 2000 complex was added to the appropriate volume of basic medium. Cells transfected to silence OCT4 were treated for 48 or 36 h for interference.

### MTT assays

Cell proliferation was monitored using a Cell Proliferation Reagent Kit I (MTT) (Roche Applied Science, Penzberg, Germany). Cells transfected with si-OCT4 (3000 cells/well) were grown in 96-well plates. Cell proliferation was assessed every 24 h following the manufacturer's protocol. All experiments were performed in quadruplicate.

### Flow cytometry analysis of apoptosis

Flow cytometry analysis was performed to determine whether suppression of OCT4 could inhibit the growth phase of cells. Cells were seeded into 6-well plates. Forty-eight hours after transfection, the cells were harvested and stained with annexin V-FITC and propidium iodide (PI), according to the manufacturer's instructions. The cellular apoptotic rate was evaluated using a FACS VerseTM flow cytometer (Becton Dickinson, CA, USA). Cells for growth phase analysis were resuspended in 200 μl PBS, fixed with 70% ice-cold ethanol overnight, and stained with PI. The cell cycle was detected by the FACSVerse™ flow cytometer.

### Statistical analysis

Student's *t*-test (two-tailed), One-way ANOVA and Mann–Whitney test were performed to analyze the data using SPSS 16.0 software. *P* values less than 0.05 were considered statistically significant.
